# Relationship between sleep quality during pregnancy and postpartum anxiety: a prospective birth cohort study

**DOI:** 10.4314/ahs.v24i3.29

**Published:** 2024-09

**Authors:** Niloofar Alizadeh, Zahra Bostani Khalesi, Fatemeh Jafarzadeh-Kenarsari, Saman Maroufizadeh

**Affiliations:** 1 Department of Midwifery, School of Nursing and Midwifery, Guilan University of Medical Sciences, Rasht, Iran; 2 Department of Biostatistics and Epidemiology, School of Health, Guilan University of Medical Sciences, Rasht, Iran

**Keywords:** Sleep, pregnant woman, postpartum period

## Abstract

The present study aimed to determine the relationship between sleep quality during pregnancy and postpartum anxiety.

A total of 237 pregnant women participated in this birth cohort study, using the sequential sampling method. The data collection tools included a demographic information form, Pittsburgh Sleep Quality Inventory, and Spielberger State-Trait Anxiety Inventory. The PSQI was completed by the participants at 28 and 40 weeks of gestation, and the STAI was completed in two stages, that is, once in the third trimester and once at eight weeks postpartum.

The mean global PSQI score of the participants was 8.11 (SD=5.41) in this study. Overall, 138 (58.2%) women had poor sleep quality. The mean score for overt anxiety was 36.58 (SD=9.37), and the mean score for latent anxiety was 37.56 (SD=9.44). There was a significant positive correlation between the total score of sleep quality during pregnancy and the scores of overt anxieties (r=0.314, P<0.001) and latent anxiety (r=0.344, P<0.001) after delivery.

The results of the present study indicated a significant relationship between sleep quality during pregnancy and postpartum anxiety. Therefore, it is recommended to evaluate the quality of sleep of pregnant women and design appropriate interventions during pregnancy to reduce postpartum anxiety.

## Introduction

Anxiety is an unpleasant emotional state, characterized by feelings of anxiety, sadness, excitement, stress, and panic 1. Postpartum anxiety is a type of anxiety disorder in women 2, which reflects their concerns about their new responsibilities and their acceptance of the new motherhood role 3. In some cases, due to the increasing severity of anxiety disorders, further evaluations and interventions are essential 4. Evidence suggests that postpartum anxiety disorder is a common mental disorder in the postpartum period, which affects 20% to 50% of young mothers 5. It is a less commonly diagnosed disorder, which may occur alone or along with depression 3. In Iran, the rate of postpartum anxiety has been estimated at 15-20% 6. It is known that postpartum anxiety can cause fatigue, low self-esteem, and reduced satisfaction with the motherhood role in women 7. Besides, if postpartum anxiety persists, it can lead to anxiety disorders, which can increase the risk of postpartum depression 8.

Numerous factors, including sleep disorders, affect postpartum psychiatric disorders 9. Sleep is an organized behavior, which is repeated as a vital necessity based on the biological rhythm 10. Sleep disorder is characterized by sleeping difficulties, poor sleep quality, and daytime dysfunction 11. The prevalence of this disorder is twice higher in women than in men, and pregnant women are more vulnerable to it compared to their non-pregnant counterparts 12. Generally, sleep disorders are one of the most common problems during pregnancy. Their prevalence varies from 13% in the early first trimester to 74% in the late third trimester 13. From almost 12 weeks of gestation until two months after delivery, pregnant women experience sleep problems, reduced sleep time, repeated late-night waking, sleeping difficulties, and disturbances in deep stages of sleep especially stages 3 and 4 which determine the quality of sleep, daytime drowsiness, excessive and frequent snoring, and breathing problems, such as obstructive sleep apnea 14. The cause of these changes is a gradual increase in the concentrations of estrogen and progesterone, which are hormones related to sleep homeostasis 15. Besides, mechanical factors, such as fetal growth, uterine contractions, abdominal pain, recurrent diuresis, leg cramps, and gastroesophageal reflux disease (GERD), negatively affect sleep quality 16.

Changes in sleep patterns during pregnancy can cause daily dysfunctions, maternal fatigue, decreased family well-being, and reduced peace of mind due to insomnia, leading to increased anxiety and fear of caring for the newborn and acceptance of the motherhood role in the family 14. Chronic sleep deprivation can negatively affect the mother-infant relationship and lead to infant neglect in severe cases. Also, sleep disorders during pregnancy, especially in the third trimester, can reduce women's pain tolerance and emotional control, increase the likelihood of cesarean section, and intensify postpartum depression 12.

According to previous studies, sleep deprivation can induce postpartum depression 15–16. Although the prevalence of anxiety is higher than postpartum depression 13, the majority of previous studies have investigated the relationship between sleep disorders and postpartum depression, while less attention has been paid to postpartum anxiety and the role of sleep disorders after childbirth. Therefore, the present study aimed to determine the relationship between sleep quality during pregnancy and postpartum anxiety.

## Methods and materials

### Study design, Sample size determination and sampling procedures

In this birth cohort study, pregnant women were selected using sequential sampling. Participants were recruited via advertisements in the Kamali Hospital, affiliated with Alborz University of Medical Sciences, Karaj, Iran. The sample size was calculated using G*Power software version 1.3. To determine the sample size, a type I error of 0.05 and a type II error of 0.2 (power, 0.8) were considered; the effect size was considered to be f2=0.10. Finally, a sample size of 237 people was selected considering a probable dropout rate of 20%.

### Inclusion criteria

The inclusion criteria were as follows: consent to participate in this study; literacy; no infertility, or no high-risk pregnancy (e.g., preeclampsia, gestational diabetes, and bleeding); no underlying or chronic diseases (according to the participant's self-report); no history of psychological disorders; no use of pills affecting sleep (according to the participant's self-report); no recent mental shock; and willingness to participate in the study.

All participants were ensured that the data collected were confidential and anonymous and they signed written consent forms.

### Data collection tools and procedures

The data collection tools included a demographic information form, Pittsburgh Sleep Quality Questionnaire (PSQI) 18, and Spielberger State-Trait Anxiety Inventory (STAI) 19. The demographic information form was a researcher-made questionnaire to determine the participants' individual and social characteristics, pregnancy characteristics (woman's age, gestational age, woman's education level, woman's occupation, spouse's age, spouse's education level, spouse's occupation, economic status, family size, number of children, and residential status [rental or home owner]), wanted or unwanted pregnancy, satisfaction with the child's sex, physical problems caused by pregnancy, income status, privacy at home, living with one's relatives or spouse's family, transportation, family conflicts, and support for taking care of the newborn. This questionnaire was completed by the researcher via interviews. The demographic information form was presented to eight faculty members to examine the face validity; after considering their recommendations, the final revisions were made.

The PSQI is a standard tool for measuring the quality and pattern of sleep. This questionnaire was designed by Buysse et al., consists of seven components, including sleep quality according to oneself, sleep latency, sleep duration, sleep efficiency, and sleep disturbances, use of sleeping medications, and daytime dysfunction in the last month. This questionnaire consists of 19 items, rated on a four-point Likert scale, and the overall score obtained from this index can range from 0 to 21. Based on the manual of the index, a score of 5 and above is defined as poor quality of sleep. The reliability coefficient of PSQI was measured to be (Cronbach's α = 0.83) 18. Internal consistency for components of the Persian version PSQI was acceptable (Cronbach's α = 0.77) 20.

The participants' anxiety was assessed using the STAI. This tool contains 40 items, 20 of which are related to overt anxiety and 20 to latent anxiety. For each scale, the items are scored on a four-point scale. In the latent anxiety section of the questionnaire, if the response indicated a lack of anxiety (i.e., items 1, 2, 5, 8, 10, 11, 15, 16, 19, and 20), it was reverse-scored (i.e., from 4 to 1). In this section of the questionnaire, there are four options for each item: “almost never”, “sometimes”, “often”, and “almost always”. Also, in the overt anxiety section of the questionnaire, there are four options for each item, scored from 1 to 4: “not at all”, “somewhat”, “moderately”, and “very much”. The scores range from 20 to 80, with higher scores indicating greater anxiety. In other words, a score of 20 represents no anxiety, and a score of 80 represents maximum anxiety. The scores of anxieties were classified as follows: normal or minimum, 20-30; mild, 31-42; moderate, 43-53; and severe, ≥54. Besides, the state anxiety scores were classified as follows: 20-34, none or minimal; 35-45, mild; 46-56, moderate; and >57, severe.

This questionnaire has been used as the most common tool to measure anxiety by various researchers, and its validity and reliability have been investigated in several studies. This tool was validated in Iran by Abdoli et al., 21 and its reliability was confirmed by internal consistency (Cronbach's alpha coefficient, 0.94). The PSQI was completed by the participants at 28 and 40 weeks of gestation, and the STAI was completed in two stages, that is, once in the third trimester and once at eight weeks postpartum.

### Ethics approval

The Guilan University of Medical Sciences review board, with the ethics code of IR.GUMS.REC.1400.076 approved the study. All methods were carried out in accordance with relevant guidelines and regulations.

### Method of data handling and analysis

In this study, quantitative variables are presented as mean and standard deviation (SD), and qualitative variables are expressed as frequency (percentage). In the univariate analyses, the relationship between postpartum anxiety scores and sleep quality during pregnancy was investigated using Pearson's correlation coefficient test. The absolute values of correlation coefficients equal to 0.1-0.3, 0.3-0.5, and >0.5 indicated low, moderate, and strong correlations, respectively. Moreover, an independent t-test was used to compare the mean scores of postpartum anxieties in mothers in terms of sleep quality during pregnancy (low and high). Cohen's effect size was also measured for the independent t-test, with d-values equal to 0.2-0.5, 0.5-0.8, and >0.8 indicating small, medium, and large effect sizes, respectively. In the multivariate analyses, to control for confounding factors, multiple linear regression was performed to identify factors related to the postpartum anxiety score. Data were analyzed usingSPSS version16, and the significance level was considered to be 0.05.

## Results

### Socioeconomic, demographic of participants

The mean age of women evaluated in this study was 27.17 (SD=5.13) years. Of 237 women, 2 (0.8%) were employed, and 126 (53.2%) had middle school or high school education (Table 1).

**Table 1:** Frequency distribution of participants according to the demographic characteristics

Variables		Frequency (percentage)
**Age (years)**	≤30	175(73.8)
	>30	62(26.2)
**Occupation**	Housewife	235(99.2)
	Employed	2(0.8)
**Education**	Elementary school	50(21.1)
	Junior high and high school	126 (53.2)
	Diploma	59(24.9)
	Academic education	2 (0.8)
**Spouse's education**	Elementary school	35 (14.8)
	Junior high and high school	154 (65.0)
	Diploma	48 (20.3)
**Spouse's occupation**	Unemployed	10 (4.2)
	Worker	88 (37.1)
	Non-governmental jobs	69 (29.1)
	Employee	64 (27.0)
	Student	6 (2.5)
**Gravidity**	G1	105 (44.3)
	G2	88 (37.1)
	≥G3	44 (18.6)
**Number of fetuses**	Singleton pregnancy	234 (98.7)
	Multiple pregnancy	3 (1.3)

The distribution of the frequency of pregnancy complications that lead to sleep disorder for the participants is shown in Table 2. Of the 237 women studied, nausea in 12 people (5.1%), abdominal bloating in 10 people (4.2%), leg cramps in 145 people (61.2%), frequent urination in 210 people (88.6) and headache in 1 person (0.4) had led to difficulty in sleeping at night. Other side effects such as heartburn, constipation and back pain did not lead to sleeping problems at night for the participants.

**Table 2:** Frequency distribution of pregnancy complications leading to sleep disorder among the participants

Pregnancy complications		Frequency (Percent)
Nausea	Yes	12(5.1)
	No	225(94.9)
Flatulence	Yes	10(4.2)
	No	227(95.8)
Heartburn	Yes	0(0)
	No	237(100)
Constipation	Yes	0(0)
	No	237(100)
Leg cramps	Yes	(61.2) 145
	No	92(38.8)
Frequent urination	Yes	210(88.6)
	No	27 (11.4)
Back ache	Yes	0(0)
	No	237(100)
Headache	Yes	1(0.4)
	No	236(99.6)

The mean global PSQI score of women was 8.11 (SD=5.41). The most common sleep disorder was sleeping latency (M=1.78). Overall, 58.2% of women had a poor sleep quality, while 41.8% had a good sleep quality (Table 3).

**Table 3:** Descriptive statistics of the participants' the PSQI total and subscale scores during pregnancy

PSQI	Mean (SD)	Median (IQR)
Subjective sleep quality	1.17 (1.08)	1 (0-2)
Sleep latency	1.78 (1.02)	2 (1-3)
Sleep duration	1.51 (0.97)	1 (1-2)
Sleep efficiency	1.00 (0.94)	1 (0-2)
Sleep disturbances	1.67 (1.03)	2 (1-3)
Use of sleeping medications	0.06 (0.25)	0 (0-0)
Daytime dysfunction	0.93 (0.98)	1 (0-2)
Global score	8.11 (5.41)	7.0 (3.5-13.0)

The participants' mean score of overt anxiety was 36.58 (SD=9.37), and the mean score of latent anxiety was 37.56 (SD=9.44). There was a significant positive correlation between the global PSQI score and the overt anxiety score (r=0.314, P<0.001) and the latent anxiety score (r=0.344, P<0.001) after delivery. The mean postpartum scores of overt anxieties (d=0.624, t (235) =4.70, P<0.001) and latent anxiety (d=0.772, t235=5.92, P<0.001) were significantly higher in women with a poor sleep quality during pregnancy compared to those with a high sleep quality during pregnancy (Table 4).

**Table 4:** Comparison of postpartum anxiety according to sleep quality during pregnancy

Variables	Groups	Mean (SD)	Mean difference	t_235_^†^	*P*	Cohen's d
**Latent anxiety**	Poor sleep quality	40.43 (8.46)	6.88	5.92	<0.001	0.772
	High sleep quality	33.56 (9.30)				
**Overt anxiety**	Poor sleep quality	38.90 (9.30)	5.56	4.70	<0.001	0.624
	High sleep quality	33.34 (8.51)				

† Independent *t*-test

The mean score of overt anxiety (M=36.58, SD=9.37) and the mean score of latent anxiety (M=37.56, SD=9.44) were significnatly higher after pregnancy compared to the mean scores (M=27.6, SD=5.7 for overt anxiety and M=30.6, SD=6.3 for latent anxiety) during pregnancy (Table 5).

**Table 5:** Descriptive statistics participants' anxiety during pregnancy and Postpartum

Variables	Possible range	Observed range	Mean (SD)	Median (IQR)
**During pregnancy**				
Overt anxiety	20-80	20-52	27.6 (5.7)	26 (23.5-30)
Latent anxiety	20-80	20-58	30.6 (6.3)	30 (26-35)
**Postpartum**				
Overt anxiety	20-80	20-59	36.58 (9.37)	36 (29-44)
Latent anxiety	20-80	20-57	37.56 (9.44)	38 (30-46)

Based on the multiple linear regression analysis, with an increase in the score of sleep quality during pregnancy, the postpartum score of overt anxiety increased. In other words, with a one-unit increase in the quality of sleep during pregnancy, the average postpartum score of overt anxiety increased by 0.52 units (b=0.52, P<0.001). Moreover, the mean postpartum score of overt anxiety in mothers with a history of abortion was significantlhigher than that of mothers without a history of abortion (b=4.07, P=0.031). Other demographic, social, and fertility characteristics of mothers had no significant relationship with the postpartum scores of overt anxieties. The coefficient of determination (R2) was equal to 0.192, which indicates that 19.2% of variance in the postpartum overt anxiety score could be explained by the variables in the model.

With an increase in the quality of sleep during pregnancy, the mothers' postpartum score of latent anxiety increased. In other words, with every one-unit increase in the quality of sleep of women during pregnancy, their postpartum score of overt anxiety increased by 0.52 units on average (b=0.52, P<0.001). Other demographic, social, and fertility characteristics of mothers had no significant relationship with the postpartum score of latent anxiety. The coefficient of determination (R2) was equal to 0.206, which indicates that 20.6% of variance in the postpartum latent anxiety scores of mothers could be explained by the variables in the model.

## Discussion

The findings of this birth cohort study indicated a significant relationship between sleep quality during pregnancy and postpartum anxiety. To the best of our knowledge, this is the first prospective study to evaluate the relationship between sleep quality and postpartum anxiety in Iran. More than 58% of participants in our study had poor sleep quality. The results of studies by Parsai et al. 22 Jomeen et al. 23, and Field et al. 5 also showed that most women had difficulty falling asleep during pregnancy. In this regard, a systematic review by Salari et al. 24 showed that the prevalence of sleep disorders in the third trimester of pregnancy was 42.4%. Studies conducted in Iran have reported similar prevalence rates in recent years (above 50%) 22-26.

In this study, frequent urination was the most common cause of sleep disturbances at night in women. In another study by Jahdi et al. 25 and Bondad et al. 27 physical complaints, such as nocturnal awakening, were the most common causes of sleep disorders in the third trimester, which is in line with the results of the current study. Many physiologic and behavioral changes occur in pregnancy that may lead to difficulties with sleep initiation or sleep maintenance 28. Pregnancy-related hormones such as estrogen, progesterone, oxytocin, and cortisol gradually increase during pregnancy, which affects sleep quality in pregnant women 13.

Besides, Physical factors such as gastro-oesophageal reflux, nausea, vomiting, heartburn, musculoskeletal pain, back pain, and spasms in the legs may also contribute to sleep disruption 9. Apart from these factors fetal movements, going to the toilet frequently at night, being unable to find a comfortable position in bed, and being unable to breathe easily are associated with increased sleep disorders during pregnancy 12,25.

The results of the present study indicated a significant positive correlation between sleep quality during pregnancy and the postpartum scores of overt and latent anxieties. The mean postpartum scores of overt and latent anxieties were significantly higher in women with poor sleep quality during pregnancy compared to women with good sleep quality. This finding is compatible with previous evidence regarding the relationship between a history of psychiatric disorders during pregnancy such as anxiety and depressive symptoms and disorders with poor sleep quality during pregnancy and the postpartum period.

Overall, changes in the quality of women's sleep, especially in the last trimester of pregnancy, can cause anxiety, depression, decreased resistance, and pain tolerance, and reduced emotional regulation 13. Iranpour et al., 29 reported a significant relationship between sleep deprivation in late pregnancy and postpartum mood disorder, which is consistent with the results of the present study. The relationship between sleep quality and postpartum anxiety may be related to arginine vasopressin, which is involved in response to stress and regulation of the sleep/wake cycle 30. One other possible mechanism could be that poor sleep quality during pregnancy causes anxiety during pregnancy and that anxiety during pregnancy can cause postpartum anxiety^31^. However, the results of a study by Okun et al. 32 in the United States contradicted these findings, as they found that sleep scores at 36 weeks of gestation did not predict postpartum anxiety; the cause of the discrepancy between the results of these studies can be the small number of samples. Besides, these studies might have evaluated the quality of sleep using different tools (e.g., Nordic Sleep Questionnaire) or might have used different criteria to define poor sleep quality.

Additionally, the postpartum score of overt anxiety in women who had a history of abortion was significantly higher than that of women without a history of abortion. Consistent with the results of the present study on the effects of abortion history on overt postpartum anxiety, several previous studies showed that experience and history of stressful events were predisposing factors for depression and postpartum anxiety 33-34. Generally, individuals are prone to anxiety when they experience events that are out of their control 3. in exposure to stressful events, people's reactions to stress vary, and the main factor in identifying stress is not its severity, but the person's specific reaction to it 5.

## Limitations

The strength of this study was its prospective design based on the community population and the relatively large sample size. On the other hand, one of the limitations of this study was the use of a self-report method to examine the quality of sleep and postpartum anxiety; it should be noted that the scores of these questionnaires only identified individuals who were exposed to poor sleep quality and anxiety. There may be other confounding factors that have not been considered in this study. In future studies, it is suggested to conduct clinical and diagnostic analyses of sleep quality and anxiety.

The results of the present study can be applied in midwifery and psychological services to improve maternal health, especially maternal sleep quality during pregnancy and anxiety and stress after childbirth.

## Conclusion

According to the findings of this study, a poor sleep quality during pregnancy was associated with latent and overt postpartum anxiety. The present findings contribute to the available evidence on the relationship between sleep quality during pregnancy and psychology during or after pregnancy. Therefore, considering the relationship between sleep disorders during pregnancy and postpartum anxiety, it is important that healthcare providers examine pregnant women for sleep disorders in order to promote an early diagnosis. Moreover, based on the present results, sleep health education and appropriate counseling during pregnancy are recommended to prevent phycological complications and promote safe pregnancies. Nevertheless, further research is required on factors that affect sleep disorders during pregnany.

## Abbreviations

PSQI: Pittsburgh Sleep Quality Inventory
STAI: Spielberger State-Trait Anxiety Inventory
IQR: interquartile range
SD: standard deviation
